# Breath Powered Nasal Delivery: A New Route to Rapid Headache Relief

**DOI:** 10.1111/head.12186

**Published:** 2013-09-11

**Authors:** Per G Djupesland, John C Messina, Ramy A Mahmoud

**Affiliations:** R&D, OptiNose US Inc.Yardley, PA, USA; R&D, OptiNose ASOslo, Norway

**Keywords:** migraine, nasal, sumatriptan, powder, drug delivery, absorption

## Abstract

The nose offers an attractive noninvasive alternative for drug delivery. Nasal anatomy, with a large mucosal surface area and high vascularity, allows for rapid systemic absorption and other potential benefits. However, the complex nasal geometry, including the narrow anterior valve, poses a serious challenge to efficient drug delivery. This barrier, plus the inherent limitations of traditional nasal delivery mechanisms, has precluded achievement of the full potential of nasal delivery. Breath Powered bi-directional delivery, a simple but novel nasal delivery mechanism, overcomes these barriers. This innovative mechanism has now been applied to the delivery of sumatriptan. Multiple studies of drug deposition, including comparisons of traditional nasal sprays to Breath Powered delivery, demonstrate significantly improved deposition to superior and posterior intranasal target sites beyond the nasal valve. Pharmacokinetic studies in both healthy subjects and migraineurs suggest that improved deposition of sumatriptan translates into improved absorption and pharmacokinetics. Importantly, the absorption profile is shifted toward a more pronounced early peak, representing nasal absorption, with a reduced late peak, representing predominantly gastrointestinal (GI) absorption. The flattening and “spreading out” of the GI peak appears more pronounced in migraine sufferers than healthy volunteers, likely reflecting impaired GI absorption described in migraineurs. In replicated clinical trials, Breath Powered delivery of low-dose sumatriptan was well accepted and well tolerated by patients, and onset of pain relief was faster than generally reported in previous trials with noninjectable triptans. Interestingly, Breath Powered delivery also allows for the potential of headache-targeted medications to be better delivered to the trigeminal nerve and the sphenopalatine ganglion, potentially improving treatment of various types of headache. In brief, Breath Powered bi-directional intranasal delivery offers a new and more efficient mechanism for nasal drug delivery, providing an attractive option for improved treatment of headaches by enabling or enhancing the benefits of current and future headache therapies.

Rapid relief in the treatment of headache is considered very important by patients, and as a result, efforts toward identifying alternative formulations and routes of delivery to improve on the speed of relief have been increasing in recent years.^1^,^2^ Although oral medications are generally preferred by most patients with headache, it is not a route of administration that is conducive to rapid action. Following oral administration, dissolution and absorption are often the rate limiting factors in determining the onset of effect. In addition, first-pass metabolism is a common barrier to the achievement of rapid therapeutic concentrations. In the case of migraine, oral delivery is often unsuitable or unattractive for many patients because of the presence of nausea and vomiting. Furthermore, migraine patients frequently have autonomic dysfunction causing delayed gastric emptying during attacks which can both delay and reduce intestinal absorption.^3^–^6^ The consequence of a slow onset of action is that the severity of the headache is prolonged and often worsening which increases the duration of disability and disease burden, and the potential to halt progression may be lost.^3^,^6^,^7^

The nose offers an attractive noninvasive alternative route of drug delivery for many conditions. Because of its large mucosal surface area and high vascularity, it allows for rapid absorption of drugs into the systemic circulation. In addition, this route of delivery may help address issues related to poor bioavailability, drug degradation in the gastrointestinal (GI) tract, and adverse events in the GI tract, and avoids the first-pass metabolism in the liver.^8^ In some instances, structures directly related to disease pathophysiology also reside in the nasal cavity, and local delivery may reduce the need for systemic exposure and improve the therapeutic index of medical treatment.^9^–^11^

A fact often ignored with nasal drug delivery is that the site of deposition within the nasal cavity highly influences the extent of systemic absorption from the nose. The anterior portions of the nose are lined with nonciliated squamous epithelium not well suited to drug absorption. However, the mucosal surface gradually transitions, as one passes deeper into the nasal cavity, to ciliated respiratory epithelium. In the regions with ciliated respiratory epithelium, branches of the ophthalmic and maxillary arteries supply the mucosal membranes covering the convoluted and complex slit-like nasal passages (Fig. 1). It is this richly vascularized region of the nasal cavity lined with respiratory epithelium which is conducive to the rapid absorption of drugs across the mucosa and into the systemic circulation from the nasal cavity and not the anterior regions lined with squamous epithelium.^8^

**Fig 1:**
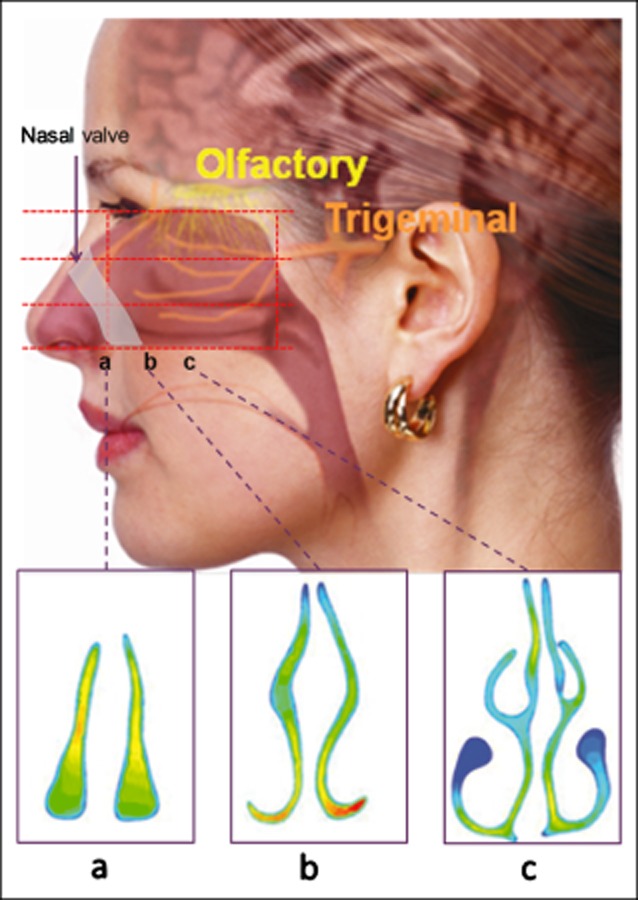
The complex anatomy of the nasal airways showing the approximate position of the olfactory and trigeminal nerves, the nasal valve, the regional segmentation (red dotted lines), and 3 different cross-sections vertical to the nasal floor at position (a), (b), and (c). The location of the nasal valve and the trigeminal and olfactory nerves.

Beyond being a route of administration for systemic delivery, there are structures within the nasal cavity that have a role in the pathophysiology of headache and may serve as therapeutic targets for headache. The trigeminal nerve, in particular the first ophthalmic branch (sensory) as well as the parasympathetic branches synapsing in the sphenopalatine ganglion (SPG), is strongly involved in the pathophysiology of cluster headache and has been implicated in migraine.^12^ Serotonin receptors (5HT_1b_ and 5HT_1d_) have been found both at the terminal nerve endings in the nasal mucosa and in the corresponding ganglia, which may offer a local target for nasally delivered drugs, triptans being only one example.^13^ Furthermore, the parasympathetic part of the trigeminal nerve may be responsible for mediating symptoms like increased tearing and nasal secretion and congestion during attacks, symptoms that are present in both cluster and migraine headache sufferers.^14^ Interestingly, levels of mediators like vasoactive intestinal peptide and calcitonin gene-related peptide (CGRP) are increased in nasal secretions and saliva during attacks, suggestive of a neurogenic inflammation in nasal vessels that potentially could enhance drug absorption across the nasal mucosa.^15^,^16^

Sinus headache, commonly associated with sinusitis, is often alleviated with the nasal administration of locally active substances including topical steroids, antihistamines, and saline. Nasal delivery of small amounts of CO_2_ for a short period of time has, in clinical trials, shown effects on migraine and on the symptoms of allergic rhinitis.^9^,^17^,^18^ It is thought that the addition of CO_2_ may induce therapeutic changes in intracellular pH, reduce CGRP release in nasal secretions, and/or produce other modifications of receptors that may mediate effects on trigeminal nerve transmission and neurogenic inflammation.^9^ Interestingly, in a recent paper, signs of nasal autonomic dysfunction with significantly higher nasal pH and reduced mucociliary clearance time were observed in Parkinson's patients compared with controls.^19^ Both exhaled nitric oxide (NO) levels and NO levels in nasal air have been found to be significantly higher in migraineurs compared with healthy volunteers. Several studies suggest a relationship between NO and neurogenic inflammation in the trigeminovascular system, which is in agreement with the observed release of various vasoactive peptides like CGRP in nasal secretions, indicating that NO may contribute negatively to the migraine disorder.^20^–^22^

## NASAL DELIVERY OF HEADACHE MEDICATIONS

Intranasal administration of a variety of medications for the treatment of headache has been utilized for some time. Intranasal formulations of dihydroergotamine mesylate (DHE), sumatriptan, zolmitriptan, butorphanol, civamide, and lidocaine have all been used/investigated for the treatment of migraine and/or cluster headache.^23^ Civamide and lidocaine have been administered via a nasal dropper to interrupt nerve transmission, and although there has been some evidence of clinical efficacy, neither has received US Food and Drug Administration approval for the treatment of headache.^24^ Furthermore, nerve stimulation of the SPG has shown promising results in aborting cluster headache, strongly supporting the potential of local treatment to nerves that may be accessed from the nasal cavity.^12^,^25^,^26^

DHE, sumatriptan, zolmitriptan, and butorphanol have obtained regulatory approval for the treatment migraine and can be administered in the form of a conventional nasal spray by the patient. DHE is known to be a highly effective medication when administered intravenously. Unfortunately, it is less than 1% bioavailable when given orally. However, when administered intranasally, it has a bioavailability of ≈40% allowing for use of this medication in the outpatient setting.^23^ In addition to the intranasal formulations, sumatriptan is available as a subcutaneous injection, an oral tablet, suppositories, and a rapid dissolving tablet (outside the United States). In addition to the intranasal formulation, zolmitriptan is available as an oral tablet and fast melt formulation. For both drugs, the intranasal formulations were introduced as alternatives to the oral formulations to overcome the issues of slow onset, reduced GI absorption during headache from slowed motility, as well as the aversion of patients to take oral medications in the presence of nausea.

Both intranasal sumatriptan and intranasal zolmitriptan have demonstrated superiority against placebo in providing relief of migraine symptoms, and intranasal zolmitriptan has been demonstrated to provide earlier relief than the same dose of zolmitriptan oral tablets.^27^–^29^ Each provides a more rapid absorption than the respective orally administered tablet.^30^ However, neither has resulted in a marked increase in total bioavailability relative to oral.^30^,^31^

These triptan conventional nasal sprays display a bimodal absorption pattern with a fairly small early peak attributed predominantly to absorption across the nasal mucosa, followed by a later more distinct peak representing GI absorption of the significant amount of drug swallowed after bypassing the nose.^30^–^33^ For zolmitriptan, the nasal fraction has been quantified in a study and found to account for approximately 30% of the total absorption.^32^ A similar study has not been conducted with sumatriptan nasal spray, though sumatriptan liquid nasal spray pharmacokinetics have been studied.^31^,^33^ It is important to note that the approved dose of zolmitriptan delivered nasally is the same as the highest dose for tablets (5 mg), whereas the range of approved conventional sumatriptan nasal spray doses (5, 10, and 20 mg) is fivefold lower than the oral doses (25, 50, and 100 mg). Consequently, the systemic exposure is significantly lower for the range of sumatriptan nasal spray doses compared with the oral formulation, whereas it is similar or even slightly higher with nasal zolmitriptan. The opportunity to deliver a lower dose highlights a potential advantage of delivering sumatriptan nasally (vs zolmitriptan) as the risk for systemic and GI-related side effects relative to the oral formulation may be reduced by lowering the systemic exposure.

Despite the theoretical advantages of intranasal drug administration, there have been impediments to broad adoption for the treatment of migraine headache. For patients, the consequences of the inadequate deposition to the target mucosa achieved with traditional nasal sprays is likely a key factor contributing to a lack of perceived clinical benefits over oral treatment. Prospective studies have demonstrated that a key driver for patients preferring a nasal spray is speed of onset. In addition, for obvious reasons, alternative formulations that offer the potential of faster absorption may be preferable over simply increasing the dose of an oral formulation.^2^,^34^ Enhanced tolerability or safety relative to oral formulations would simply add to patient preference should they accompany a core efficacy benefit like improved speed of onset.

## LIMITATIONS OF NASAL SPRAY DELIVERY

Traditional spray pumps used with nasal sprays result in limited drug deposition to the target sites beyond the narrow triangular-shaped constriction called the nasal valve, which is located approximately 2 cm from the entrance of the nostril.^35^,^36^ The purpose of the narrow nasal valve, in concert with the complex convoluted nasal passageways, is to filter and condition the inspired air, enhance olfaction, and optimize gas exchange and fluid retention during exhalation.^8^ These important functional features of the nose impose important limitations on efficient nasal drug delivery that are too often ignored.

For example, the expanding convex spray plume and high particle speed emitted from a spray bottle will largely impact on the walls of the nasal vestibule. Increasing the propulsive force of the nasal delivery does not alter the fundamental anatomic constraints, as the plume impacts on the first surfaces it reaches, while “sniffing” exacerbates the problem as described later. The anterior segment of the nasal cavity, the nasal vestibule, is lined primarily with nonciliated squamous epithelium, which is less efficient for medication absorption than the ciliated respiratory epithelium beyond the nasal valve^8^ (Fig. 1). Because of this mismatch between the geometry of the anterior region of the nose and the spray plume, only a small fraction of the spray penetrates beyond the nasal valve, and a large portion of the spray volume remains in the vestibule.

The large volume of liquid in the vestibule of the nose may drip out or be wiped off. Sniffing during delivery further narrows the nasal valve, and reflexive sniffing after delivery to avoid drip-out will not only further narrow the nasal valve, which is already particularly narrow superiorly (see Fig. 1a), but also shrink the already slit-like deeper nasal passages (Fig. 1b,c). This tends to impair both the intended targeting to a broad nasal surface area and any potential benefits of higher deposition, and tends to direct whatever medication penetrates the nasal valve along the nasal floor to be swallowed. Taste buds sensing bitter taste located at the base of the tongue are quickly exposed to the concentrated liquid that contributes to the intense bitter taste often reported with these nasal sprays. It is only the smaller proportion of the spray that reaches the highly vascularized respiratory mucosa that accounts for most of the early nasal absorption. Such a significant portion of the medication delivered by conventional nasal sprays is swallowed, rather than being nasally absorbed, which the GI tract contributes more to the amount of drug absorbed than does the nose.^31^,^33^ This phenomenon is clearly observed with sumatriptan where a bimodal absorption profile is produced following conventional nasal spray administration: a lower early peak, likely related to intranasal absorption, is produced after 20 minutes and is followed by a higher absorption peak consistent with GI absorption around 90 minutes.^31^

The predominance of oral absorption following conventional nasal spray delivery reduces the intended advantages of nasal delivery. Thus, the lack of significant differentiation from oral tablets results in only marginally faster onset of action in some patients and likely contributes to the limited uptake in the market place observed with nasal sprays.

Notably, both the sensory and parasympathetic branches of the trigeminal nerve involved in the pathophysiology of migraine and other headaches innervate the mucosal surfaces beyond the nasal valve, which is also where the SPG resides. To the extent that these structures are involved in headache pathophysiology, the posterior and superior portion of the nasal cavity would be an interesting target for therapeutic intervention with current or future drugs; however, they cannot be effectively reached with a standard nasal spray (Fig. 1).

## OPTINOSE BREATH POWERED DELIVERY

### Breath Powered Mechanism of Action and Devices

A comprehensive review on deposition patterns associated with nasal drops and spray pumps concluded that traditional delivery devices are suboptimal for delivery to the respiratory mucosa beyond the nasal valve.^8^ Several approaches attempting to improve the drug delivery of traditional spray pumps have been suggested and tested over the years, but are generally either impractical, suboptimal, or have yet to be proven in replicated human intranasal deposition studies. Efforts to optimize conventional nasal sprays by improving the method of use have been similarly unrewarding: a study tested 7 different head and body positions using traditional nasal sprays and concluded that there is “no best method.”^37^

The Breath Powered Bi-Directional delivery mechanism can be implemented in simple devices without electromechanical cost or complexity, and overcomes many deficiencies of traditional nasal delivery. Both liquid and powder drugs can be delivered using such devices, and implementations of each are in active development. This novel nasal delivery concept consists of devices with a flexible mouthpiece and a shaped, sealing nosepiece. It is designed to exploit unique aspects of the nasal anatomy and physiology to improve the extent and reproducibility of drug delivery to target sites in the nose beyond the nasal valve while avoiding the risk of lung inhalation.^38^

The user slides the shaped nosepiece into one nostril to create a seal with the nasal tissue, inserts the mouthpiece between the open lips, takes a deep breath, closes the lips around the mouthpiece, and then exhales forcefully into the mouthpiece. The oral exhalation into the device creates a positive pressure in the oropharynx, naturally elevating and sealing the soft palate and completely separating the nasal and oral cavities. Because of the sealing nosepiece, the airflow and dynamic positive pressure is transferred by the device into the nasal cavity where it expands the nasal valve and narrow slit-like passages. The intranasal pressure, which is slightly reduced compared with the intraoral driving pressure due to the resistance of the device and the nasal passage, automatically balances the pressure across the soft palate to avoid overelevation of the soft palate. This is essential as it maintains patency of the important communication pathway between the two nostrils that is located deep in the nasal cavity posterior to the nasal septum, permitting the exhaled breath to escape from the contralateral nostril while relieving the nasal cavity of excess pressure.

**Fig 2:**
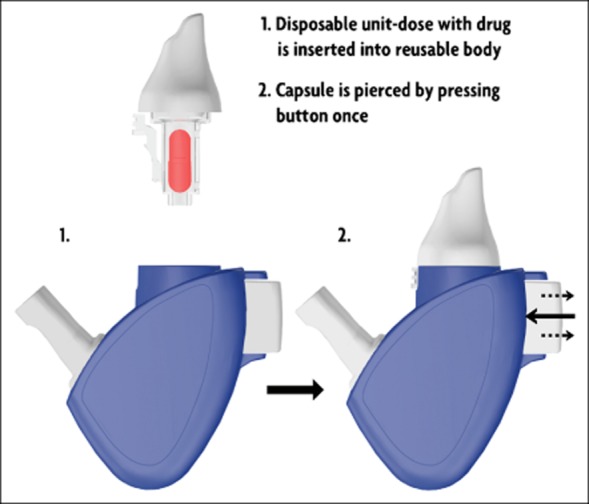
The Breath Powered powder device for sumatriptan delivery.

**Fig 3:**
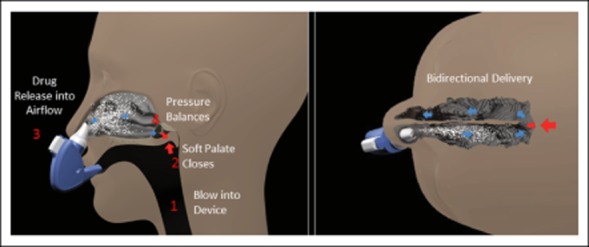
Illustration of the Breath Powered nasal delivery.

A dedicated multiuse Breath Powered powder device with a reusable device body and a disposable nosepiece was developed for use in patients with migraine headache (Fig. 2). An 11-mg dose of sumatriptan powder is filled into a standard respiratory capsule and provided to the patient in a capsule chamber of a disposable nosepiece. There is a small entrance for airflow at the bottom of the chamber and a larger opening at the top. Prior to use of the device, a fresh nosepiece is snapped into the top of the device, and the capsule is pierced by depressing a button on the device body. Upon exhalation into the device, the pierced capsule vibrates and rotates with the exhaled breath, releasing the powder into the airflow. Drug particles are carried posteriorly by the expanding flow of physiologically warmed air into one nostril, beyond the nasal valve, and deposited broadly throughout the deep nasal cavity before the air reverses course and escapes anteriorly through the other nostril (Bi-directional delivery) (Fig. 3).

Multiple studies evaluating anthropometric differences between individuals were conducted in order to develop the appropriate design of the device in order to accommodate differences in individual nostril size and distances and angles between the mouth and nose. The current design has been found in usability testing as well as clinical trials to be well accepted in terms of comfort and ease of use.

### Drug Deposition With Breath Powered Devices

The scintigraphic techniques used in the last decades to study *in vivo* nasal deposition of liquid and powder formulations are relatively crude and did not allow for reliable absolute or relative quantification of regional nasal deposition and clearance patterns. An improved system allowing reliable quantification of the regional nasal deposition of radiolabeled particles in human subjects has been introduced and used in clinical deposition trials comparing conventional nasal spray devices to Breath Powered devices for both liquid and powder drugs.^39^

**Fig 4:**
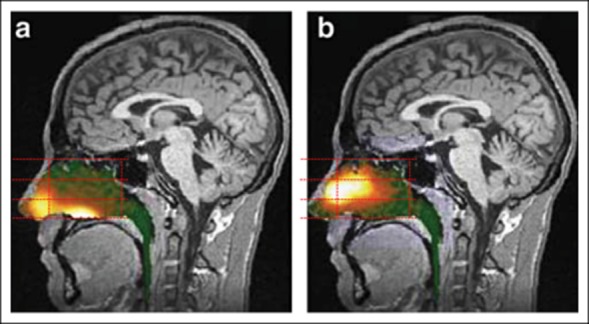
Gamma camera images 2 minutes after delivery using a traditional liquid spray (a) and powder with OptiNose Breath Powered Device (b) shown with a logarithmic hot iron intensity scale. Initial gamma images from one of the subjects are superimposed on a lateral magnetic resonance (MR) image. The red dotted lines indicate the segmentation used for regional quantification.

In the most recent study, Tc^99m^-labeled lactose powder was delivered with the Breath Powered powder device.^35^ A capsule fill and particle size profile similar to sumatriptan powder was used. For measuring differences in deposition, the nose was divided into 3 horizontal segments, and a vertical dividing line was positioned at the head of the inferior turbinate (Fig. 4), and radiation counts within each segment were quantified after administration.

**Fig 5:**
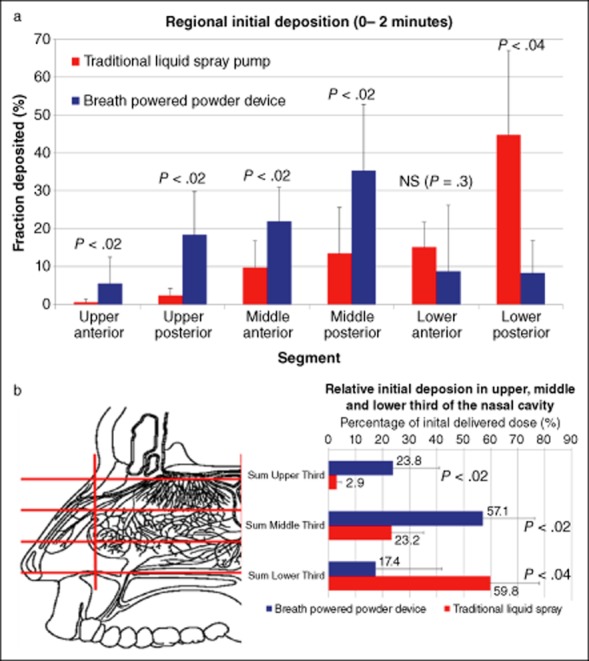
(a) Initial Regional nasal deposition (0-2 minutes) for Breath Powered powder delivery device and delivery with a traditional nasal spray pump. (b) Initial horizontal nasal distribution (0-2 minutes) for the Breath Powered powder delivery device and delivery with a conventional nasal spray pump.

The Breath Powered powder device demonstrated a broader deposition on the regions where nasal mucosa is lined by ciliated respiratory epithelium (especially upper and middle posterior regions, but also the upper anterior and middle anterior regions) with less deposition in the nonciliated nasal vestibule and significantly greater initial deposition to the upper posterior regions beyond the nasal valve compared with the conventional spray delivery (≈54% vs 16%) (Figs. 4 and 5a).^35^ In contrast, liquid sprays deposited most of the dose (≈60% vs ≈17%) in limited regions in the lower parts of the nose (Fig. 5).^35^

### Pharmacokinetics (PK) of Breath Powered Sumatriptan Nasal Powder

The regional analyses of deposition and clearance clearly demonstrate that the Breath Powered powder device provides broader exposure to the highly vascularized respiratory mucosa beyond the nasal valve, and particularly improves delivery to the middle and upper regions of the nasal cavity.^35^ This should reasonably be expected to translate into more rapid and more extensive drug absorption of suitable medications than is achieved with standard nasal spray delivery. This difference should be possible to measure objectively, as it should be reflected in improved PK and ultimately in improved efficacy. Such studies have now been performed assessing the consequences of delivering sumatriptan in this fashion.^40^,^41^

**Fig 6:**
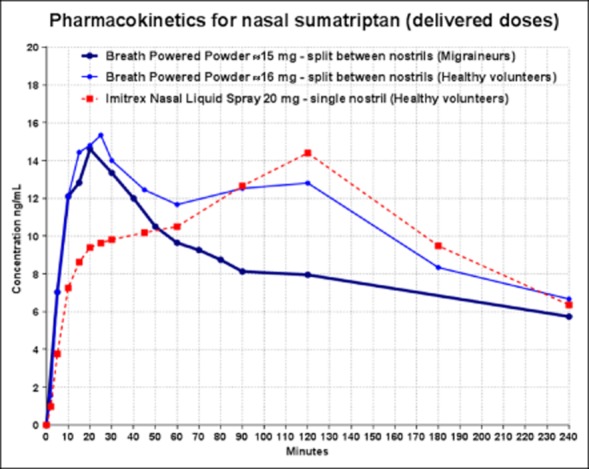
Pharmacokinetics (PK) profiles for nasal sumatriptan from two crossover studies performed with the Breath Powered powder device and the marketed Imitrex sumatriptan nasal spray. The one study was done in migraine patients during GTN-challenge, whereas the other study was performed in healthy volunteers.

Two studies have evaluated the PK of sumatriptan delivered with the OptiNose Breath Powered device. One was a crossover study in 12 migraine patients pretreated with either subcutaneous (SC) injection sumatriptan, or sumatriptan powder delivered with a Breath Powered device, prior to a challenge with nitroglycerine known to induce migraine (GTN-challenge).^40^ The larger second study was a 4-way crossover study in healthy volunteers comparing sumatriptan powder delivered with a Breath Powered device (15 mg delivered dose split between nostrils) to 20 mg sumatriptan nasal spray (1 nostril), 100 mg sumatriptan tablet, and 6 mg sumatriptan SC injection.^41^ In both studies, there was a bimodal absorption pattern representing an initial nasal absorption followed by a GI absorption with Breath Powered delivery (Fig. 6).^40^,^41^ The initial peak observed in both studies was more pronounced than the peak observed with the standard nasal spray (as measured in the second study),^41^ indicative along with other PK parameters of a more efficient and faster systemic absorption with the Breath Powered device (Fig. 6). Absorption also occurred earlier than with tablet delivery but with a significantly lower peak and total systemic exposure than either the oral tablet or subcutaneous injection.^41^

The nasal peak for sumatriptan powder is very similar in the two PK studies, one in migraineurs and one in healthy volunteers, occurring early in both populations.^40^,^41^ However, the later peak, assumed to represent predominantly GI absorption, is substantially smaller in the study performed in migraineurs during GTN-challenge^40^ (Fig. 6). This likely reflects the delayed and decreased GI absorption because of autonomic dysfunction observed in migraineurs that is further accentuated during an attack.^3^–^6^

It should be noted that sumatriptan powder was split between the two nostrils while the nasal spray was administered to a single nostril.^40^,^41^ The impact on the PK profile of dividing the liquid spray dose between nostrils has been previously investigated and found not to improve either the rate or extent of absorption over administration to a single nostril.^42^ Therefore, it seems unlikely that this difference in administration procedure explains the findings in the PK study in healthy subjects.

It is important to recall when reviewing the pharmacokinetic data that the total delivered sumatriptan dose with the Breath Powered delivery device is 20-25% lower than the sumatriptan 20 mg liquid spray. A shift to greater nasal absorption with Breath Powered delivery reduces the fraction of sumatriptan bypassing the nose compared with sumatriptan spray, and the dose is split between the two nostrils (Fig. 6). The lower dose, broader nasal distribution, and significantly altered clearance pattern (NB, the soft palate is closed at the time of delivery) following Breath Powered delivery further reduce the amount and concentration of drug reaching the taste buds at the base of the tongue, which is likely to mitigate the intensity of the bitter taste sensation. The results show that the enhanced nasal deposition produced by the Breath Powered device is indeed associated with pharmacokinetic advantages.

### Clinical Trials in Migraine

It is reasonable to hypothesize that the increased early absorption may offer advantages in terms of improved efficacy and in particular more rapid onset of pain relief, and that the low dose may enhance tolerability or safety. The ability to prevent migraine attacks in the study with GTN-challenge combined with the similar electroencephalography findings following SC and Breath Powered powder delivery, despite much lower blood levels, also suggest potential clinically relevant advantages.^40^ These findings provided the rationale to proceed to a randomized placebo-controlled trial with a Breath Powered sumatriptan delivery device.

In the first placebo-controlled, parallel group, 3-arm trial in acute migraine (117 total patients), two doses of sumatriptan powder were delivered with the Breath Powered device and compared with a “placebo” control group using dummy devices.^43^ Fast onset of pain relief was observed for both active doses with early pain relief rates similar to historical data for SC injection despite much lower systemic exposure. Significant benefits were also observed for pain relief at 120 minutes for both doses, and the higher dose was selected for further development. The higher dose produced a response of 80% vs 44% with placebo (*P* < .01) at 2 hours, and high early response rates at 60 minutes (74% vs 38%, *P* < .01) and at 30 minutes (54% vs 31%; NS).^43^

A phase III, placebo-controlled, parallel group, 2-arm study in 212 patients was recently conducted with sumatriptan powder being delivered with the Breath Powered device.^44^ At 2 hours postdose, a significant proportion of patients experienced pain relief compared with placebo (68% vs 45%, *P* < .01), a high value for triptan therapy. However, again, the most striking result was the fast onset of pain relief, with a remarkably high response rate at 30 minutes (42% vs 27%, *P* < .05). This is particularly notable in light of the extremely low dose of a triptan medication. The reported adverse events were primarily mild and transient and generally limited to the site of administration. It was concluded that Breath Powered delivery of intranasal sumatriptan powder is effective, safe, and well tolerated and can offer fast onset of pain relief in adults with acute migraine headache.

Comparison of these results with published data suggest that the speed of onset of pain relief is much faster than oral treatment and approaches that achieved with SC injection, but with substantially lower systemic exposure and therefore the attendant risk of adverse events.^44^

### Potential Therapeutic Effects of the Breath Powered Device

In each clinical trial with Breath Powered delivery, an interestingly high placebo response rate has been observed.^43^,^44^ In these trials, control patients did not receive “no treatment” but used identical Breath Powered delivery devices to active patients. Although the high response among these “placebo” patients may be due to chance, secular trends, or other factors, it is interesting to note that there are also potential explanations directly relating to the use of the Breath Powered device.

During normal respiration, there is minimal exchange of air in the upper narrow part of the nose. The particular aerodynamics of the Breath Powered delivery device blowing a large amount of exhaled air with 5-6% CO_2_ at a flow rate of 30 L/minute or more lasting for 2-3 seconds, which penetrates the upper narrow segments of the nose, could provide therapeutic effects, in part similar to those reported with the delivery of 100% CO_2_, albeit that this CO_2_ delivery was done for short duration and done at low flow (10 mL/s) and low volume.^17^,^18^ In the present Breath Powered device, it is postulated that the oscillating capsule and airflow may significantly enhance exchange of air in upper narrow parts of the nose, as in part observed in response to humming and pulsating nebulizers.^45^,^46^ In addition, there are reasons to hypothesize that potential positive effects mediated by the positive air pressure, rapid vibrations produced by the rattling capsule, and the removal of NO may all play a role in alleviating migraine headache.^46^,^47^ One or more of these, or other, device-related mechanisms may contribute to the high response rate in the placebo groups in the trials with Breath Powered powder delivery in migraine patients.

### Potential Headache Treatments With Breath Powered Bi-Directional Delivery

The deep nasal cavity deposition associated with Breath Powered delivery enables the potential for medications to be delivered more broadly to the trigeminal nerve innervated tissue and to the SPG, which may prove to be beneficial in the treatment of a range of headache disorders. The aerodynamic properties of the device itself may offer alternative mechanisms of action and/or synergetic effects.

In addition to possibilities in preemption or prevention of migraine, cluster headache and trigeminal neuralgia represent target indications for possible delivery of numerous new or current drugs alone or in combination, including for example triptans, DHE, lidocaine, nonsteroidal anti-inflammatory drugs (NSAIDs), locally acting corticosteroids, and potentially CGRP-antagonists. There is great unmet need, and it is possible to modify the current device to optimize delivery for treatments intended to particularly target the region closest to the SPG for optimal efficacy. Other potential indications include chronic migraine, where delivery of a very small daily dose of a triptan or other drugs in this manner may offer sufficient receptor blockage to reduce the number of acute attacks. Even topical steroids may prove valuable alone or as an adjuvant therapy in cluster headache or in sinus headache.

## CONCLUSIONS

Nasal drug delivery has long been a route of administration known to be useful in the treatment of headache and other disorders. However, the typical methods of intranasal delivery are relatively ineffective in their delivery of medication broadly and to the posterior/superior areas of the nasal cavity where rapid and efficient drug absorption and other benefits can effectively accrue. Therefore, the promise of intranasal drug delivery has not been fully realized. Human gamma-deposition studies *in vivo* with Breath Powered devices have proven that this novel device mechanism is capable of producing a significantly improved nasal drug deposition pattern. Pharmacokinetic studies to assess the consequences of this improved deposition were performed following the delivery of a low dose of sumatriptan powder, and show that this improved delivery is associated with enhanced speed and efficiency of absorption across the nasal mucosa with a reduced proportion of GI absorption relative to standard nasal spray. In replicated clinical trials, Breath Powered delivery of low-dose sumatriptan has now been shown to produce substantial response rates, with early pain relief more similar to SC injection than to other forms of delivery, but with much lower exposure than with oral or SC treatment. This new form of nasal delivery may offer a number of interesting therapeutic options for the treatment of a range of headache disorders in the future.

## References

[1] Davies GM, Santanello N, Lipton R (2000). Determinants of patient satisfaction with migraine therapy. Cephalalgia.

[2] Dowson A, Bundy M, Salt R, Kilminster S (2007). Patient preference for triptan formulations: A prospective study with zolmitriptan. Headache.

[3] Aurora SK, Papapetropoulos S, Kori SH, Kedar A, Abell TL (2013). Gastric stasis in migraineurs: Etiology, characteristics, and clinical and therapeutic implications. Cephalalgia.

[4] Thomsen LL, Dixon R, Lassen LH (1996). 311C90 (Zolmitriptan), a novel centrally and peripheral acting oral 5-hydroxytryptamine-1D agonist: A comparison of its absorption during a migraine attack and in a migraine-free period. Cephalalgia.

[5] Sramek JJ, Hussey EK, Clements B, Cutler NR (1999). Oral sumatriptan pharmacokinetics in the migraine state. Clin Drug Investig.

[6] Ferrari A, Pinetti D, Bertolini A, Coccia C, Sternieri E Interindividual variability of oral sumatriptan pharmacokinetics and of clinical response in migraine patients. Eur J Clin Pharmacol.

[7] Mathew R, Andreou AP, Chami L (2011). Immunohistochemical characterization of calcitonin gene-related peptide in the trigeminal system of the familial hemiplegic migraine 1 knock-in mouse. Cephalalgia.

[8] Djupesland PG (2013). Nasal drug delivery devices: Characteristics and performance in a clinical perspective-a review. Drug Deliv Transl Res.

[9] Vause C, Bowen E, Spierings E, Durham P (2007). Effect of carbon dioxide on calcitonin gene-related peptide secretion from trigeminal neurons. Headache.

[10] Goadsby PJ Sphenopalatine (pterygopalatine) ganglion stimulation and cluster headache: New hope for ye who enter here. Cephalalgia.

[11] Schoenen J, Vandersmissen B, Jeangette S (2013). Migraine prevention with a supraorbital transcutaneous stimulator: A randomized controlled trial. Neurology.

[12] Schoenen J, Jensen RH, Lanteri-Minet M (2013). Stimulation of the sphenopalatine ganglion (SPG) for cluster headache treatment. Pathway CH-1: A randomized, sham-controlled study. Cephalalgia.

[13] Ivanusic JJ, Kwok MM, Jennings EA (2011). Peripheral targets of 5-HT(1D) receptor immunoreactive trigeminal ganglion neurons. Headache.

[14] Barbanti P, Fabbrini G, Pesare M, Vanacore N, Cerbo R (2002). Unilateral cranial autonomic symptoms in migraine. Cephalalgia.

[15] Cady RK, Vause CV, Ho TW, Bigal ME, Durham PL (2009). Elevated saliva calcitonin gene-related peptide levels during acute migraine predict therapeutic response to rizatriptan. Headache.

[16] Bellamy JL, Cady RK, Durham PL (2006). Salivary levels of CGRP and VIP in rhinosinusitis and migraine patients. Headache.

[17] Casale TB, Romero FA, Spierings EL (2008). Intranasal noninhaled carbon dioxide for the symptomatic treatment of seasonal allergic rhinitis. J Allergy Clin Immunol.

[18] Spierings E (2005). Abortive treatment of migraine headache with non-inhaled, intranasal carbon dioxide: A randomized, double-blind, placebo-controlled, parallel-grpup study. Headache.

[19] Kotan D, Tatar A, Aygul R, Ulvi H (2013). Assessment of nasal parameters in determination of olfactory dysfunction in Parkinson's disease. J Int Med Res.

[20] Van der Schueren BJ, Verbrugge FH, Verbesselt R, Van Hecken A, Depre M, de Hoon JN (2010). No arguments for increased endothelial nitric oxide synthase activity in migraine based on peripheral biomarkers. Cephalalgia.

[21] Olesen J, Thomsen LL, Lassen LH, Olesen IJ (1995). The nitric oxide hypothesis of migraine and other vascular headaches. Cephalalgia.

[22] Messlinger K, Lennerz JK, Eberhardt M, Fischer MJ (2012). CGRP and NO in the trigeminal system: Mechanisms and role in headache generation. Headache.

[23] Rapoport AM, Bigal ME, Tepper SJ, Sheftell FD (2004). Intranasal medications for the treatment of migraine and cluster headache. CNS Drugs.

[24] Maizels M, Geiger AM (1999). Intranasal lidocaine for migraine: A randomized trial and open-label follow-up. Headache.

[25] Kudrow L, Kudrow DB, Sandweiss JH (1995). Rapid and sustained relief of migraine attacks with intranasal lidocaine: Preliminary findings. Headache.

[26] Hardebo JE, Elner A (1987). Nerves and vessels in the pterygopalatine fossa and symptoms of cluster headache. Headache.

[27] Ryan R, Elkind A, Baker CC, Mullican W, DeBussey S, Asgharnejad M (1997). Sumatriptan nasal spray for the acute treatment of migraine. Results of two clinical studies. Neurology.

[28] Gawel M, Aschoff J, May A, Charlesworth BR (2005). Zolmitriptan 5 mg nasal spray: Efficacy and onset of action in the acute treatment of migraine – results from phase 1 of the REALIZE Study. Headache.

[29] Charlesworth BR, Dowson AJ, Purdy A, Becker WJ, Boes-Hansen S, Farkkila M (2003). Speed of onset and efficacy of zolmitriptan nasal spray in the acute treatment of migraine: A randomised, double-blind, placebo-controlled, dose-ranging study versus zolmitriptan tablet. CNS Drugs.

[30] Goadsby PJ, Yates R (2006). Zolmitriptan intranasal: A review of the pharmacokinetics and clinical efficacy. Headache.

[31] Duquesnoy C, Mamet JP, Sumner D, Fuseau E (1998). Comparative clinical pharmacokinetics of single doses of sumatriptan following subcutaneous, oral, rectal and intranasal administration. Eur J Pharm Sci.

[32] Kagedal M, Zingmark PH, Hedlund C, Yates R (2005). True nasopharyngeal absorption of zolmitriptan after administration via nasal spray in healthy. Am J Drug Deliv.

[33] Fuseau E, Petricoul O, Moore KH, Barrow A, Ibbotson T (2002). Clinical pharmacokinetics of intranasal sumatriptan. Clin Pharmacokinet.

[34] Fox AW (2004). Onset of effect of 5-HT1B/1D agonists: A model with pharmacokinetic validation. Headache.

[35] Djupesland PG, Skretting A (2012). Nasal deposition and clearance in man: Comparison of a bidirectional powder device and a traditional liquid spray pump. J Aerosol Med Pulm Drug Deliv.

[36] Aggarwal R, Cardozo A, Homer JJ (2004). The assessment of topical nasal drug distribution. Clin Otolaryngol Allied Sci.

[37] Merkus P, Ebbens FA, Muller B, Fokkens WJ (2006). Influence of anatomy and head position on intranasal drug deposition. Eur Arch Otorhinolaryngol.

[38] Djupesland PG, Skretting A, Winderen M, Holand T (2004). Bi-directional nasal delivery of aerosols can prevent lung deposition. J Aerosol Med.

[39] Skretting A, Djupesland PG (2009). A new method for scintigraphic quantification of deposition and clearance in anatomical regions of the human nose. Nucl Med Commun.

[40] Luthringer R, Djupesland PG, Sheldrake CD (2009). Rapid absorption of sumatriptan powder and effects on glyceryl trinitrate model of headache following intranasal delivery using a novel bi-directional device. J Pharm Pharmacol.

[41] Obaidi M, Offman E, Messina J, Carothers J, Djupesland P, Mahmoud R (2013). Improved pharmacokinetics of sumatriptan with Breath Powered™ nasal delivery of sumatriptan powder. Headache.

[42] Salonen R, Ashford E, Dahlof C (1994). Intranasal sumatriptan for the acute treatment of migraine. International Intranasal Sumatriptan Study Group. J Neurol.

[43] Djupesland PG, Docekal P (2010). Intranasal sumatriptan powder delivered by a novel breath-actuated bi-directional device for the acute treatment of migraine: A randomised, placebo-controlled study. Cephalalgia.

[44] Cady R, Messina J, Carothers J, Mahmoud R (2013). Efficacy and safety of a novel Breath-Powered™ Powder sumatriptan intranasal treatment for acute migraine. Headache.

[45] Moller W, Schuschnig U, Khadem Saba G (2010). Pulsating aerosols for drug delivery to the sinuses in healthy volunteers. Otolaryngol Head Neck Surg.

[46] Eby GA (2006). Strong humming for one hour daily to terminate chronic rhinosinusitis in four days: A case report and hypothesis for action by stimulation of endogenous nasal nitric oxide production. Med Hypotheses.

[47] Suren M, Kaya Z, Ozkan F, Erkorkmaz U, Arici S, Karaman S (2013). Comparison of the use of the Valsalva maneuver and the eutectic mixture of local anesthetics (EMLA((R))) to relieve venipuncture pain: A randomized controlled trial. J Anesth.

